# Health Sciences before, during and after the COVID-19 Pandemic

**DOI:** 10.3390/ejihpe13040057

**Published:** 2023-04-06

**Authors:** Mihnea-Alexandru Găman

**Affiliations:** 1 Faculty of Medicine, “Carol Davila” University of Medicine and Pharmacy, 8 Eroii Sanitari Boulevard, 050474 Bucharest, Romania; mihneagaman@yahoo.com; 2 Department of Hematology, Center of Hematology and Bone Marrow Transplantation, Fundeni Clinical Institute, 258 Fundeni Road, 022328 Bucharest, Romania

The COVID-19 pandemic has heavily influenced the teaching and practical training required for students enrolled in health sciences courses globally both at undergraduate, graduate and postgraduate levels. This public health threat has impacted not only the formation of physicians, dentists, pharmacists, nurses and midwives but of all healthcare professionals, mainly due to the cancellation of clinical clerkships and the transition of on-site to online education [1,2]. However, many students have experienced increased levels of anxiety, depression, fatigue, substance abuse, and less physical exercise following online training, or have even considered a career switch to non-healthcare-related fields. Moreover, a significant proportion of doctors-to-be have admitted that virtual courses cannot accurately replace or simulate hospital rotations and the interaction of real patients and consider that the lack of this clinical training might also reflect on their decision to pursue a certain specialty following residency [3,4,5]. However, students did have the opportunity to be actively engaged and volunteer in public hospitals to help end the COVID-19 pandemic and acquire indispensable abilities for future physicians [6]. 

Thus, it is beyond doubt that a Special Issue dedicated to the COVID-19 pandemic was warranted in the *European Journal of Investigation in Health, Psychology and Education*. 

Herein, in the Special Issue *Health Sciences before, during and after the COVID-19 Pandemic*, we explored relevant topics regarding the impact of the SARS-CoV-2 pandemic on (medical) education, as well as highlighted the results of research endeavors that thrived during this period of isolation. Following a thorough external peer-review, five papers were accepted for publication in this Special Issue: two original articles, one brief report and two systematic reviews (one of which also contained a meta-analysis).

In their cross-sectional study, Rogowska and Meres assessed the crosstalk established between job and life satisfaction and emotional intelligence. Their investigation enrolled over 300 subjects, consisting mainly of primary and secondary school teachers and pinpointed that emotional intelligence predicts both life and job satisfaction. In addition, they demonstrated that job satisfaction predicts life satisfaction, concluding that job satisfaction emerges as a mediator in the crosstalk of emotional intelligence and life satisfaction [7].

Nash evaluated the potential role of doodling in the evaluation of burnout in healthcare researchers who experienced anxiety and/or depression related to their workplace activities. The investigator compared in-person pre-pandemic versus online meetings during the COVID-19 pandemic and pointed out that individuals who partook in these mindfulness activities were more likely to feel relaxed when doodling during in-person meetings, possibly because there was more interaction between participants during physical meetings [8].

Tanoubi et al. explored the benefits of simulation-based education in the training of dental surgeons in procedural sedation and the management of related complications by means of theoretical and practical applications. A total of 16 Canadian dentists with expertise in oral and maxillofacial surgery were trained in anesthesia techniques and crisis resource management using simulation-based clinical scenarios. The authors argue for a need for sedation training for dental surgeons engaging in oromaxillofacial interventions [9].

As the COVID-19 pandemic has mostly impacted the production of original research, researchers worldwide have focused on the production of narrative or systematic reviews, as well as meta-analyses, two of which have been published in this Special Issue.

In their systematic review, Tsagkaris et al. investigated the applications of infrared radiation techniques in the management of musculoskeletal conditions: knee osteoarthritis, fibromyalgia, chronic low back pain, chronic myofascial syndrome, sacroiliitis and Gulf War Illness. Based on their findings, infrared radiation has been successfully applied in the therapeutic approach of knee osteoarthritis, fibromyalgia and chronic myofascial syndrome [10].

In addition, Moysidis et al. explored the interplay between polycystic ovary syndrome and atrial fibrillation based on the data derived from electrocardiographic and echocardiographic studies. Their findings reflect that polycystic ovary syndrome is characterized by abnormal atrial conduction, as the meta-analysis point out that there is a prolonged maximum P-wave duration, an increased P-wave dispersion, increased echocardiographic measurements of atrial electromechanical delay, as well as altered electromechanical coupling parameters in the aforementioned endocrine disorder [11].

In conclusion, the present Special Issue provides an overview of several aspects of the impact of the COVID-19 pandemic on health sciences, and also allows for the publication of several high-quality papers.
